# 2-Meth­oxy-*N*-[5-(2-methoxy­phen­yl)-1,3,4-thia­diazol-2-yl]benzamide hemi­hydrate

**DOI:** 10.1107/S1600536808019934

**Published:** 2008-07-31

**Authors:** Li-he Yin, Rong Wan, Feng Han, Bin Wang, Jin-tang Wang

**Affiliations:** a Department of Applied Chemistry, College of Science, Nanjing University of Technology, No. 5 Xinmofan Road, Nanjing 210009, People’s Republic of China

## Abstract

In the mol­ecule of the title compound, C_17_H_15_N_3_O_3_S·0.5H_2_O, the thia­diazole ring is oriented with respect to the two 2-methoxy­phenyl rings at dihedral angles of 3.70 (3) and 1.74 (2)°. An intra­molecular N—H⋯O hydrogen bond results in the formation of a planar six-membered ring, which is oriented with respect to the thia­diazole ring at a dihedral angle of 1.33 (3)°. Thus, all of the rings are nearly coplanar. In the crystal structure, inter­molecular O—H⋯N and C—H⋯O hydrogen bonds link the mol­ecules.

## Related literature

For related literature, see: Nakagawa *et al.* (1996 ▶); Wang *et al.* (1999 ▶).
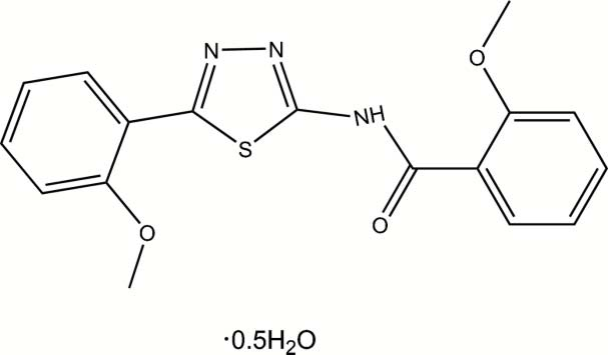


## Experimental

### 

#### Crystal data

C_17_H_15_N_3_O_3_S·0.5H_2_O
*M* *_r_* = 350.40
Monoclinic, 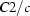

*a* = 29.950 (6) Å
*b* = 14.561 (3) Å
*c* = 7.6520 (15) Å
β = 94.78 (3)°
*V* = 3325.4 (12) Å^3^
*Z* = 8
Mo *K*α radiation
μ = 0.22 mm^−1^
*T* = 298 (2) K
0.30 × 0.10 × 0.05 mm

#### Data collection

Enraf–Nonius CAD-4 diffractometer
Absorption correction: ψ scan (North *et al.*, 1968 ▶) *T*_min_ = 0.937, *T*_max_ = 0.989
6340 measured reflections
3003 independent reflections
1602 reflections with *I* > 2σ(*I*)
*R*_int_ = 0.060
3 standard reflections frequency: 120 min intensity decay: none

#### Refinement

*R*[*F* ^2^ > 2σ(*F* ^2^)] = 0.079
*wR*(*F* ^2^) = 0.240
*S* = 1.04
3003 reflections
222 parameters
H-atom parameters constrained
Δρ_max_ = 0.57 e Å^−3^
Δρ_min_ = −0.33 e Å^−3^

### 

Data collection: *CAD-4 Software* (Enraf–Nonius, 1989 ▶); cell refinement: *CAD-4 Software*; data reduction: *XCAD4* (Harms & Wocadlo, 1995 ▶); program(s) used to solve structure: *SHELXS97* (Sheldrick, 2008 ▶); program(s) used to refine structure: *SHELXL97* (Sheldrick, 2008 ▶); molecular graphics: *PLATON* (Spek, 2003 ▶); software used to prepare material for publication: *SHELXTL* (Sheldrick, 2008 ▶).

## Supplementary Material

Crystal structure: contains datablocks global, I. DOI: 10.1107/S1600536808019934/hk2483sup1.cif

Structure factors: contains datablocks I. DOI: 10.1107/S1600536808019934/hk2483Isup2.hkl

Additional supplementary materials:  crystallographic information; 3D view; checkCIF report

## Figures and Tables

**Table 1:** Hydrogen-bond geometry (Å, °)

*D*—H⋯*A*	*D*—H	H⋯*A*	*D*⋯*A*	*D*—H⋯*A*
N3—H3*A*⋯O3	0.86	2.04	2.706 (5)	134
O4—H4⋯N1^i^	0.85	2.24	2.796 (6)	123
C14—H14*A*⋯O2^ii^	0.93	2.49	3.316 (6)	148

Symmetry codes: (i) 
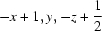
; (ii) 
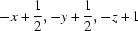
.

**Fig. 1.**
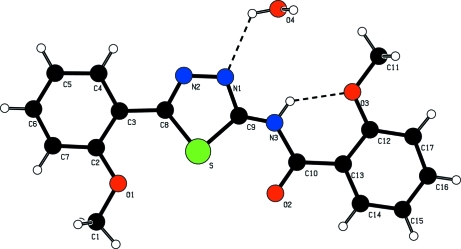
The molecular structure of the title molecule, with the atom-numbering scheme. Hydrogen bonds are shown as dashed lines.

**Fig. 2.**
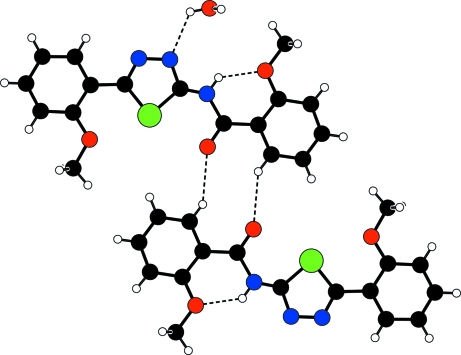
A partial packing diagram of the title compound. Hydrogen bonds are shown as dashed lines.

## supplementary crystallographic information

### Comment

1,3,4-Thiadiazole derivatives represent an interesting class of compounds possessing broad spectrum biological activities (Nakagawa *et al.*, 1996; Wang *et al.*,  1999). These compounds are known to exhibit diverse biological effects, such as insecticidal and fungicidal activities (Wang *et al.*, 1999). We report herein the crystal structure of the title compound.

In the molecule of the title compound (Fig. 1) the bond lengths and angles are within normal ranges. Rings A (C2-C7), B (S/N1/N2/C8/C9) and C (C12-C17) are, of course, planar. The intramolecular N-H···O hydrogen bond (Table 1) results in the formation of a six-membered planar ring D (N3/H3A/O3/C10/C12/C13). The dihedral angles between the rings are A/B = 3.70 (3)°, A/C = 2.81 (3)°, A/D = 2.45 (3)°, B/C = 1.74 (2)°, B/D = 1.33 (3)° and C/D = 0.90 (3)°. So, all of the rings are nearly coplanar.

In the crystal structure, intermolecular O-H···N and C-H···O hydrogen bonds (Table 1) link the molecules (Fig. 2), in which they may be effective in the stabilization of the structure.

### Experimental

For preparation of the title compound, a solution of 5-(2-methoxyphenyl) -1,3,4-thiadiazol-2-amine (5 mmol) in pyridine (50 ml) was cooled to 273 K. To this solution, 2-methoxybenzoyl chloride (5 mmol) was added via a drop funnel over period of 30 min. The mixture was stirred at 273 K for 1 h, raised to room temperature and reacted for 1 h. The pyridine was distilled and the solid residue was recrystallized from ethanol to give the title compound (m.p. 513-514 K). Crystals suitable for X-ray analysis were obtained by slow evaporation of an acetone solution.

### Refinement

H atoms were positioned geometrically, with O-H = 0.85 Å (for H_2_O), N-H = 0.86 Å (for NH) and C-H = 0.93 and 0.96 Å for aromatic and methyl H, respectively, and constrained to ride on their parent atoms with U_iso_(H) = xU_eq_(C,N,O), where x = 1.5 for methyl H and x = 1.2 for all other H atoms.

### Crystal data

**Table:**

C_17_H_15_N_3_O_3_S·0.5H_2_O	*F*_000_ = 1464
*M**_r_* = 350.40	*D*_x_ = 1.400 Mg m^−^^3^
Monoclinic, *C*2/*c*	Melting point = 513–514 K
Hall symbol: -C 2yc	Mo *K*α radiation λ = 0.71073 Å
*a* = 29.950 (6) Å	Cell parameters from 25 reflections
*b* = 14.561 (3) Å	θ = 9–12º
*c* = 7.6520 (15) Å	µ = 0.22 mm^−^^1^
β = 94.78 (3)º	*T* = 298 (2) K
*V* = 3325.4 (12) Å^3^	Block, colorless
*Z* = 8	0.30 × 0.10 × 0.05 mm

### Data collection

**Table:**

Enraf–Nonius CAD-4 diffractometer	*R*_int_ = 0.061
Radiation source: fine-focus sealed tube	θ_max_ = 25.2º
Monochromator: graphite	θ_min_ = 1.4º
*T* = 298(2) K	*h* = −35→35
ω/2θ scans	*k* = 0→17
Absorption correction: ψ scan(North *et al.*, 1968)	*l* = 0→9
*T*_min_ = 0.937, *T*_max_ = 0.989	3 standard reflections
6340 measured reflections	every 120 min
3003 independent reflections	intensity decay: none
1602 reflections with *I* > 2σ(*I*)	

### Refinement

**Table:**

Refinement on *F*^2^	Secondary atom site location: difference Fourier map
Least-squares matrix: full	Hydrogen site location: inferred from neighbouring sites
*R*[*F*^2^ > 2σ(*F*^2^)] = 0.079	H-atom parameters constrained
*wR*(*F*^2^) = 0.241	*w* = 1/[σ^2^(*F*_o_^2^) + (0.1018*P*)^2^ + 4.918*P*] where *P* = (*F*_o_^2^ + 2*F*_c_^2^)/3
*S* = 1.04	(Δ/σ)_max_ < 0.001
3003 reflections	Δρ_max_ = 0.57 e Å^−^^3^
222 parameters	Δρ_min_ = −0.33 e Å^−^^3^
Primary atom site location: structure-invariant direct methods	Extinction correction: none

### Special details

**Table:**

Geometry. All e.s.d.'s (except the e.s.d. in the dihedral angle between two l.s. planes) are estimated using the full covariance matrix. The cell e.s.d.'s are taken into account individually in the estimation of e.s.d.'s in distances, angles and torsion angles; correlations between e.s.d.'s in cell parameters are only used when they are defined by crystal symmetry. An approximate (isotropic) treatment of cell e.s.d.'s is used for estimating e.s.d.'s involving l.s. planes.
Refinement. Refinement of F^2^ against ALL reflections. The weighted R-factor wR and goodness of fit S are based on F^2^, conventional R-factors R are based on F, with F set to zero for negative F^2^. The threshold expression of F^2^ > 2sigma(F^2^) is used only for calculating R-factors(gt) etc. and is not relevant to the choice of reflections for refinement. R-factors based on F^2^ are statistically about twice as large as those based on F, and R- factors based on ALL data will be even larger.

### Fractional atomic coordinates and isotropic or equivalent isotropic displacement parameters (Å^2^)

**Table:**

	*x*	*y*	*z*	*U*_iso_*/*U*_eq_	
S	0.14065 (4)	0.20610 (9)	0.03340 (17)	0.0643 (5)	
O1	0.16810 (11)	0.1136 (2)	−0.2572 (5)	0.0694 (10)	
O2	0.19106 (10)	0.2584 (3)	0.3483 (5)	0.0761 (11)	
O3	0.09246 (10)	0.3994 (2)	0.5803 (4)	0.0623 (9)	
O4	1.0000	0.3791 (4)	0.2500	0.139 (3)	
H4	0.9969	0.3526	0.3472	0.167*	
N1	0.06625 (14)	0.2824 (4)	0.0904 (6)	0.0805 (14)	
N2	0.06140 (12)	0.2445 (3)	−0.0693 (5)	0.0622 (11)	
N3	0.11780 (9)	0.3022 (2)	0.3039 (5)	0.0430 (8)	
H3A	0.0969	0.3324	0.3490	0.052*	
C1	0.20716 (19)	0.0758 (5)	−0.3250 (9)	0.105 (2)	
H1B	0.2323	0.0820	−0.2392	0.157*	
H1C	0.2132	0.1081	−0.4299	0.157*	
H1D	0.2022	0.0120	−0.3516	0.157*	
C2	0.12874 (16)	0.1123 (3)	−0.3594 (6)	0.0575 (12)	
C3	0.09255 (15)	0.1571 (3)	−0.2910 (6)	0.0543 (11)	
C4	0.05153 (18)	0.1584 (4)	−0.3904 (7)	0.0796 (16)	
H4A	0.0273	0.1894	−0.3501	0.095*	
C5	0.0471 (2)	0.1124 (5)	−0.5522 (8)	0.0946 (19)	
H5A	0.0194	0.1107	−0.6165	0.114*	
C6	0.0828 (2)	0.0707 (5)	−0.6157 (8)	0.0954 (19)	
H6A	0.0794	0.0422	−0.7248	0.115*	
C7	0.1229 (2)	0.0696 (4)	−0.5235 (7)	0.0742 (15)	
H7A	0.1470	0.0403	−0.5690	0.089*	
C8	0.09539 (14)	0.2027 (3)	−0.1186 (6)	0.0515 (11)	
C9	0.10742 (16)	0.2703 (4)	0.1660 (8)	0.0652 (14)	
C10	0.16044 (14)	0.2990 (3)	0.4122 (6)	0.0509 (11)	
C11	0.05722 (17)	0.4453 (4)	0.6622 (8)	0.0873 (19)	
H11A	0.0303	0.4444	0.5846	0.131*	
H11B	0.0658	0.5077	0.6873	0.131*	
H11C	0.0520	0.4143	0.7694	0.131*	
C12	0.13322 (14)	0.3932 (3)	0.6683 (6)	0.0486 (10)	
C13	0.16640 (13)	0.3438 (3)	0.5879 (5)	0.0467 (10)	
C14	0.20858 (15)	0.3341 (3)	0.6785 (6)	0.0599 (12)	
H14A	0.2305	0.3006	0.6276	0.072*	
C15	0.21853 (18)	0.3729 (4)	0.8408 (7)	0.0683 (14)	
H15A	0.2467	0.3653	0.9000	0.082*	
C16	0.1862 (2)	0.4227 (4)	0.9133 (7)	0.0790 (17)	
H16A	0.1934	0.4515	1.0205	0.095*	
C17	0.14394 (18)	0.4321 (3)	0.8354 (7)	0.0653 (13)	
H17A	0.1223	0.4639	0.8917	0.078*	

### Atomic displacement parameters (Å^2^)

**Table:**

	*U*^11^	*U*^22^	*U*^33^	*U*^12^	*U*^13^	*U*^23^
S	0.0561 (8)	0.0660 (8)	0.0708 (8)	0.0122 (6)	0.0047 (6)	−0.0030 (7)
O1	0.064 (2)	0.077 (2)	0.069 (2)	0.0236 (17)	0.0142 (18)	−0.0027 (19)
O2	0.0527 (19)	0.107 (3)	0.068 (2)	0.0223 (19)	0.0017 (16)	−0.028 (2)
O3	0.0505 (18)	0.070 (2)	0.067 (2)	0.0199 (15)	0.0081 (16)	−0.0046 (18)
O4	0.095 (5)	0.093 (5)	0.239 (10)	0.000	0.060 (5)	0.000
N1	0.069 (3)	0.105 (4)	0.067 (3)	0.022 (3)	0.006 (2)	−0.011 (3)
N2	0.051 (2)	0.087 (3)	0.047 (2)	0.018 (2)	−0.0025 (17)	−0.011 (2)
N3	0.0212 (15)	0.0347 (18)	0.074 (2)	0.0118 (13)	0.0067 (16)	−0.0007 (18)
C1	0.091 (4)	0.124 (5)	0.104 (5)	0.055 (4)	0.040 (4)	0.011 (4)
C2	0.071 (3)	0.047 (3)	0.054 (3)	0.002 (2)	0.004 (2)	0.000 (2)
C3	0.060 (3)	0.055 (3)	0.048 (2)	−0.003 (2)	0.001 (2)	−0.003 (2)
C4	0.067 (3)	0.102 (4)	0.066 (3)	−0.004 (3)	−0.014 (3)	−0.004 (3)
C5	0.096 (4)	0.122 (5)	0.062 (3)	−0.019 (4)	−0.021 (3)	−0.015 (3)
C6	0.129 (5)	0.088 (4)	0.067 (4)	−0.011 (4)	−0.006 (3)	−0.016 (3)
C7	0.106 (4)	0.058 (3)	0.060 (3)	0.008 (3)	0.016 (3)	−0.011 (3)
C8	0.054 (3)	0.048 (3)	0.052 (2)	0.003 (2)	0.003 (2)	0.000 (2)
C9	0.054 (3)	0.069 (3)	0.073 (3)	0.015 (3)	0.013 (3)	0.015 (3)
C10	0.047 (2)	0.049 (3)	0.055 (3)	0.006 (2)	−0.003 (2)	0.003 (2)
C11	0.070 (3)	0.101 (5)	0.095 (4)	0.042 (3)	0.025 (3)	0.002 (4)
C12	0.050 (2)	0.044 (2)	0.051 (2)	0.0060 (19)	0.006 (2)	0.004 (2)
C13	0.045 (2)	0.048 (2)	0.046 (2)	−0.0029 (19)	0.0030 (19)	−0.004 (2)
C14	0.045 (2)	0.067 (3)	0.067 (3)	−0.001 (2)	0.002 (2)	−0.003 (3)
C15	0.069 (3)	0.074 (4)	0.059 (3)	−0.011 (3)	−0.012 (3)	−0.004 (3)
C16	0.102 (4)	0.064 (3)	0.067 (3)	−0.014 (3)	−0.021 (3)	−0.009 (3)
C17	0.084 (3)	0.059 (3)	0.055 (3)	0.004 (3)	0.020 (3)	−0.009 (2)

### Geometric parameters (Å, °)

**Table:**

S—C8	1.712 (4)	C4—C5	1.404 (8)
S—C9	1.751 (6)	C4—H4A	0.9300
O1—C2	1.360 (5)	C5—C6	1.354 (9)
O1—C1	1.429 (6)	C5—H5A	0.9300
O2—C10	1.226 (5)	C6—C7	1.343 (8)
O3—C12	1.347 (5)	C6—H6A	0.9300
O3—C11	1.436 (5)	C7—H7A	0.9300
O4—H4	0.8501	C10—C13	1.492 (6)
N1—C9	1.329 (6)	C11—H11A	0.9600
N1—N2	1.337 (5)	C11—H11B	0.9600
N2—C8	1.271 (5)	C11—H11C	0.9600
N3—C9	1.171 (6)	C12—C13	1.409 (6)
N3—C10	1.464 (5)	C12—C17	1.411 (7)
N3—H3A	0.8600	C13—C14	1.397 (6)
C1—H1B	0.9600	C14—C15	1.374 (7)
C1—H1C	0.9600	C14—H14A	0.9300
C1—H1D	0.9600	C15—C16	1.364 (8)
C2—C7	1.398 (7)	C15—H15A	0.9300
C2—C3	1.404 (6)	C16—C17	1.361 (7)
C3—C4	1.390 (6)	C16—H16A	0.9300
C3—C8	1.472 (6)	C17—H17A	0.9300
			
C8—S—C9	87.3 (2)	N2—C8—S	113.1 (3)
C2—O1—C1	118.8 (4)	C3—C8—S	127.2 (3)
C12—O3—C11	118.8 (4)	N3—C9—N1	120.5 (5)
C9—N1—N2	111.7 (4)	N3—C9—S	127.8 (4)
C8—N2—N1	116.1 (4)	N1—C9—S	111.7 (4)
C9—N3—C10	130.6 (4)	O2—C10—N3	115.8 (4)
C9—N3—H3A	114.7	O2—C10—C13	122.2 (4)
C10—N3—H3A	114.7	N3—C10—C13	122.0 (4)
O1—C1—H1B	109.5	O3—C11—H11A	109.5
O1—C1—H1C	109.5	O3—C11—H11B	109.5
H1B—C1—H1C	109.5	H11A—C11—H11B	109.5
O1—C1—H1D	109.5	O3—C11—H11C	109.5
H1B—C1—H1D	109.5	H11A—C11—H11C	109.5
H1C—C1—H1D	109.5	H11B—C11—H11C	109.5
O1—C2—C7	124.0 (5)	O3—C12—C13	117.3 (4)
O1—C2—C3	116.0 (4)	O3—C12—C17	123.6 (4)
C7—C2—C3	120.0 (5)	C13—C12—C17	119.0 (4)
C4—C3—C2	118.5 (4)	C14—C13—C12	118.4 (4)
C4—C3—C8	117.8 (4)	C14—C13—C10	116.0 (4)
C2—C3—C8	123.7 (4)	C12—C13—C10	125.5 (4)
C3—C4—C5	119.4 (6)	C15—C14—C13	121.8 (5)
C3—C4—H4A	120.3	C15—C14—H14A	119.1
C5—C4—H4A	120.3	C13—C14—H14A	119.1
C6—C5—C4	120.7 (6)	C16—C15—C14	118.6 (5)
C6—C5—H5A	119.6	C16—C15—H15A	120.7
C4—C5—H5A	119.6	C14—C15—H15A	120.7
C7—C6—C5	121.0 (6)	C17—C16—C15	122.8 (5)
C7—C6—H6A	119.5	C17—C16—H16A	118.6
C5—C6—H6A	119.5	C15—C16—H16A	118.6
C6—C7—C2	120.4 (5)	C16—C17—C12	119.3 (5)
C6—C7—H7A	119.8	C16—C17—H17A	120.4
C2—C7—H7A	119.8	C12—C17—H17A	120.4
N2—C8—C3	119.6 (4)		
			
C9—N1—N2—C8	1.8 (7)	N2—N1—C9—N3	177.2 (5)
C1—O1—C2—C7	5.2 (7)	N2—N1—C9—S	−1.8 (6)
C1—O1—C2—C3	−174.9 (5)	C8—S—C9—N3	−177.8 (5)
O1—C2—C3—C4	179.6 (4)	C8—S—C9—N1	1.1 (4)
C7—C2—C3—C4	−0.5 (7)	C9—N3—C10—O2	2.0 (7)
O1—C2—C3—C8	−0.8 (7)	C9—N3—C10—C13	−179.0 (5)
C7—C2—C3—C8	179.1 (4)	C11—O3—C12—C13	176.9 (4)
C2—C3—C4—C5	2.2 (8)	C11—O3—C12—C17	−2.3 (7)
C8—C3—C4—C5	−177.4 (5)	O3—C12—C13—C14	−178.2 (4)
C3—C4—C5—C6	−3.0 (10)	C17—C12—C13—C14	1.0 (6)
C4—C5—C6—C7	2.0 (11)	O3—C12—C13—C10	1.1 (6)
C5—C6—C7—C2	−0.3 (10)	C17—C12—C13—C10	−179.7 (4)
O1—C2—C7—C6	179.4 (5)	O2—C10—C13—C14	−2.1 (7)
C3—C2—C7—C6	−0.5 (8)	N3—C10—C13—C14	179.0 (4)
N1—N2—C8—C3	178.5 (4)	O2—C10—C13—C12	178.6 (4)
N1—N2—C8—S	−0.9 (6)	N3—C10—C13—C12	−0.3 (7)
C4—C3—C8—N2	−2.9 (7)	C12—C13—C14—C15	−1.3 (7)
C2—C3—C8—N2	177.5 (5)	C10—C13—C14—C15	179.3 (4)
C4—C3—C8—S	176.4 (4)	C13—C14—C15—C16	−0.7 (8)
C2—C3—C8—S	−3.2 (7)	C14—C15—C16—C17	3.1 (9)
C9—S—C8—N2	−0.2 (4)	C15—C16—C17—C12	−3.5 (8)
C9—S—C8—C3	−179.5 (4)	O3—C12—C17—C16	−179.5 (4)
C10—N3—C9—N1	−179.7 (4)	C13—C12—C17—C16	1.3 (7)
C10—N3—C9—S	−0.9 (8)		

### Hydrogen-bond geometry (Å, °)

**Table:**

*D*—H···*A*	*D*—H	H···*A*	*D*···*A*	*D*—H···*A*
N3—H3A···O3	0.86	2.04	2.706 (5)	134
O4—H4···N1^i^	0.85	2.24	2.796 (6)	123
C14—H14A···O2^ii^	0.93	2.49	3.316 (6)	148

Symmetry codes: (i) −*x*+1, *y*, −*z*+1/2; (ii) −*x*+1/2, −*y*+1/2, −*z*+1.
